# Comparative Analysis of the In Vitro Fermentation Characteristics of Polysaccharides from *Polygonatum cyrtonema* Hua with Different Growth Years Using Human Fecal Microbiota

**DOI:** 10.3390/foods15172954

**Published:** 2026-08-22

**Authors:** Rongguang Yang, Luning Zhao, Ying Zhu, Yansheng Zhao, Juan Bai, Xiang Xiao

**Affiliations:** School of Food Science and Engineering, Jiangsu University, Zhenjiang 212013, China; 13409194403@163.com (R.Y.); 1000005022@ujs.edu.cn (L.Z.); ying307@126.com (Y.Z.); zhaoys@ujs.edu.cn (Y.Z.)

**Keywords:** *Polygonatum cyrtonema* Hua, polysaccharide, in vitro fermentation, antioxidant activities, gut microbiota, short-chain fatty acid

## Abstract

*Polygonatum cyrtonema* Hua polysaccharides (PCPs) are bioactive components with antioxidant and immunomodulatory properties. However, whether the polysaccharides derived from elder *Polygonatum cyrtonema* exhibit superior health benefits remains unclear. This study systematically compared the in vitro fermentation characteristics, gut microbiota-modulating effects, and short-chain fatty acid (SCFA) production of PCPs extracted from three-year-old (TPP), five-year-old (FPP), and eight-year-old (EPP) plants using a human fecal fermentation model. The fermentation dynamics were evaluated by monitoring pH, OD_600_, and the consumption of total and reducing sugars, while the structural degradation and compositional differences in PCPs were tracked via molecular weight distribution and monosaccharide composition analysis. The results showed that all three PCPs were degraded and utilized by gut microbiota, accompanied by decreased pH, increased OD_600_, and enhanced antioxidant activities. High-throughput 16S rDNA sequencing revealed that, at the phylum level, all PCPs increased the Firmicutes/Bacteroidetes ratio. At the genus level, they reduced the abundance of harmful bacteria such as *Sutterella* and increased beneficial bacteria including *Bifidobacterium* and *Megasphaera*. Furthermore, gas chromatography (GC) analysis demonstrated that, compared with FPP, TPP and EPP significantly promoted the production of SCFAs. In summary, this study indicates that the in vitro fermentation characteristics and prebiotic properties of PCPs vary with growth years, and a comprehensive evaluation suggests that three-year-old *Polygonatum cyrtonema* Hua represents a promising raw material for the development of functional foods.

## 1. Introduction

*Polygonatum cyrtonema* Hua (*P. cyrtonema* Hua) is a perennial herb belonging to the genus *Polygonatum* within the family Liliaceae [1]. As the primary edible part of the genus *Polygonatum*, the rhizome possesses anti-fatigue effects and is traditionally used to nourish Lung Yin [2,3]. Studies have shown that the rhizome of *P. cyrtonema* Hua contains various bioactive components, such as polysaccharides, steroidal saponins, flavonoids, alkaloids and amino acids [4], in which *P. cyrtonema* Hua polysaccharide (PCP) is among the main active compounds. PCP is a heteropolysaccharide, including fructose, glucose, galactose, mannose, and arabinose [5]. Many studies have confirmed that PCP exhibits multiple biological activities, such as antioxidation [6], hypoglycemic effects [7], anti-inflammatory effects [8], and anti-fatigue effects [9]. Its mechanism of action involves regulating cytokine secretion, eliminating free radicals, inhibiting inflammatory signaling pathways, and improving the structure of the intestinal flora [10,11,12,13].

As the plant growth period extends, the accumulation patterns of metabolites in the roots and rhizomes undergo alterations. Such changes may consequently influence the structural characteristics and fermentation properties of polysaccharides [14,15]. For instance, in *Polygonatum odoratum* (Mill.) Druce, the contents of most saccharides serving as precursors for polysaccharide synthesis in the rhizomes were significantly higher in two-year-old plants than in three-year-old plants, and the accumulation levels of steroidal saponins, amino acids, their derivatives, and alkaloids were also greater in two-year-old plants compared with three-year-old plants [16]. To date, research on the differences in physicochemical properties and in vitro fermentation characteristics of PCPs of different growth years remains limited, which is of critical importance for selecting the appropriate growth year of *P. cyrtonema* Hua rhizomes as raw materials for functional food development.

In vitro fermentation models simulate the anaerobic environment of the human colon by utilizing fecal microbiota from healthy volunteers to ferment the tested polysaccharides [17,18,19]. Through the determination of changes in total sugar and reducing sugar contents, short-chain fatty acids (SCFAs) production, pH value, and antioxidant activity, the extent of polysaccharide utilization by gut microbiota and its potential health benefits can be comprehensively evaluated. Yonggan Sun et al. investigated the effects of *Aloe gel polysaccharide* on gut microbiota following in vitro fermentation, and demonstrated that this polysaccharide promoted SCFAs production while inhibiting branched-chain fatty acids (BCFAs) and indole, and exerted prebiotic effects by enriching *Faecalibacterium* and *Parabacteroides* [20]. Most plant polysaccharides are resistant to direct hydrolysis by digestive enzymes in the human gastrointestinal tract and can reach the colon in a relatively intact form [21,22,23], where they serve as fermentable carbon sources for gut microbiota and exert prebiotic effects [24,25,26]. Studies have demonstrated that simulated salivary treatment does not alter the chemical properties or structural characteristics of PCPs; only a minor proportion of PCPs undergoes degradation via glycosidic bond cleavage in simulated gastric and intestinal fluids. Owing to the absence of endogenous enzymes capable of degrading PCPs in the human digestive tract, PCPs can reach the colon in a relatively intact form, where they are subsequently degraded and utilized by the gut microbiota, thereby exerting prebiotic effects [27].

Therefore, this study aimed to systematically investigate the in vitro physicochemical properties and biological activities of PCP from three-year-old (TPP), five-year-old (FPP), and eight-year-old (EPP) plants. A particular focus was placed on their roles in SCFA production and regulation of the intestinal microbiota with in vitro fermentation models. Through this research, the effect of growth duration on the fermentation characteristics of PCP was clarified. These findings provided a solid experimental and theoretical basis for developing probiotic products based on growth duration and for applying them in functional foods.

## 2. Materials and Methods

### 2.1. Materials and Reagents

*Polygonatum cyrtonema* Hua (Qingyang, Anhui, China). ABTS, DPPH, SCFA standards, galacturonic acid and carbazole (Shanghai Aladdin Biochemical Technology Co., Ltd., Shanghai, China). BCA kit and total antioxidant capacity assay kit (T-AOC) (Nanjing Jiancheng Bioengineering Institute, Nanjing, China). Other regents used in this study (Sinopharm Chemical Reagent Co., Ltd., Shanghai, China).

### 2.2. Extraction of PCPs and Determination of the Component Contents

An optimized hot water extraction-ethanol precipitation method was adopted for extracting TPP, FPP and EPP, with the procedures as follows: dried *P. cyrtonema* Hua was ground and defatted using 75% ethanol, then distilled water was added, subjected to 90 °C water bath for 2.5 h, centrifuged to obtain the supernatants, which was repeated three times. The combined supernatants were concentrated by a rotary evaporator. Sevag method was applied for deproteinization, followed by adding 95% ethanol 4 °C overnight to obtain the precipitate. After being dissolved in distilled water, the precipitate was dialyzed for 3 days, and freeze-dried under vacuum for 48 h to obtain the extracts. Contents of total sugar, glucuronic acid and protein were determined using the phenol-sulfuric acid method [28], carbazole sulfate method [29] and BCA kit, respectively.

### 2.3. In Vitro Fermentation by Human Fecal Microbiota

In vitro fermentation was performed as described by Songtao Fan et al. [30]. M2GSC medium was prepared according to a published method [31]. One liter of medium contained 2.0 g peptone, 4.0 g yeast extract, 0.02 g hemin, 0.46 g L-cysteine, 0.5 g bile salts, 0.1 g NaCl, 0.04 g KH_2_PO_4_, 0.04 g K_2_HPO_4_, 0.01 g CaCl_2_, 0.01 g MgSO_4_, 2 g NaHCO_3_, 2 mL resazurin solution (0.025%, *w*/*v*), 2 mL Tween 80, and 0.01 g vitamin K. The medium was sterilized at 121 °C for 30 min.

Fresh human feces were collected from three healthy donors (two males and one female, aged 20–30 years). All donors had a regular diet, no history of gastrointestinal disease, and no antibiotic use in the past three months; they also voluntarily signed the informed consent form. The study was conducted in accordance with the Declaration of Helsinki, and approved by the Institutional Review Board (or Ethics Committee) of Jiangsu University (protocol code JSDX20260330008 and date of approval: 30 March 2026). Fecal samples from the three donors were pooled, then mixed with saline at a ratio of 1:10 (*w*/*v*). The mixture was centrifuged at 500 rpm for 5 min, and the supernatant was used as the fecal inoculum.

Each Hungate anaerobic tube was filled with 5.5 mL of M2GSC medium, 4.5 mL of fecal inoculum, and 60 mg of PCP under CO_2_ flow. The tubes were sealed and incubated at 37 °C under anaerobic conditions. A blank control without PCP was also prepared. At 0, 6, 12, 24, and 48 h, 1 mL of fermentation slurry was collected under CO_2_ flow, placed in sterile tubes, and stored at −80 °C. Each time point had three replicates.

Gas production was measured by volumetric displacement using sterile syringes. The pH was determined with an electrochemical test pen. OD_600_ was measured using a microplate reader. Reducing sugar content was determined by the dinitrosalicylic acid (DNS) method.

### 2.4. Analysis of Molecular Weight, Infrared and Monosaccharide Composition of PCPs After In Vitro Fermentation

#### 2.4.1. Molecular Weight Determination

The molecular weight of PCPs was determined by high-performance size exclusion chromatography coupled with multi-angle laser light scattering detector (HPSEC-MALLS), conducted at 25 °C using the Agilent 1100 HPLC system (Agilent, Santa Clara, CA, USA) with two SEC columns (Shodex OHpak SB-806 HQ and SB-805 HQ, 8 × 300 mm, Shodex, Minato-ku, Tokyo, Japan). The mobile phase was ultrapure water and the flow rate was 0.5 mL/min, column temperature was 25 °C and the injection volume was 10 μL [32]. Dextran standards with different molecular weights (1 mg/mL) were used to construct the standard curve, and the molecular weight of the PCPs (1 mg/mL) was analyzed after filtration.

#### 2.4.2. FT-IR Spectroscopy

FT-IR spectra of the freeze-dried PCPs fermentation broths were processed using the OMNIC 8.2 software package. Prior to sample measurement, a background spectrum was collected with the empty sample compartment. The freeze-dried polysaccharide samples were then placed on the sample stage along the optical path, and FT-IR spectra were recorded using a Fourier transform infrared spectrometer over the wavenumber range of 4000–500 cm^−1^. The transmittance spectra and corresponding data tables were obtained, and the final FT-IR spectra were plotted using Origin 2024 [33].

#### 2.4.3. Monosaccharide Composition Analysis

The monosaccharide composition of PCPs was analyzed by high-performance anion-exchange chromatography with pulsed amperometric detection (HPAEC-PAD) on an Agilent 1100 instrument (Agilent, Santa Clara, CA, USA). The sample preparation was conducted according to Xiao et al. [34]. The chromatographic column was Agilent ZORBAX Eclipse XDB-C18 (4.6 × 250 mm), the mobile phase was 83% 0.05 M KH_2_PO_4_ + 17% C_2_H_3_N, the flow rate was 1 mL/min, the injection volume was 5 μL, and the column temperature was 30 °C.

### 2.5. Determination of Antioxidant Activity After In Vitro Fermentation

The ABTS radical scavenging activity of each sample was assayed following the method described by Zheng et al. [35]. The ABTS radical scavenging rate was calculated using the formula below:

(1) $$ABTS\;radical\;scavenging\;rate\;(\%)=\left[1-\left(A_{1}-A_{2}\right)/A_{0}\right]$$

wherein A_1_ denoted the absorbance of the fermentation broth and ABTS+ solution at 734 nm; A_2_ denoted the absorbance of the fermentation broth and ethanol at 734 nm; and A_0_ denoted the absorbance of the ethanol and ABTS+ solution at absorbance value at 734 nm.

The DPPH radical scavenging activity of each sample was determined according to the method reported by Li et al. [36]. The DPPH radical scavenging rate was calculated with the following formula:

(2) $$DPPH\;radical\;scavenging\;rate\;(\%)=\left[1-\left(A_{1}-A_{2}\right)/A_{0}\right]$$

wherein A_1_ represents the absorbance of the fermentation broth and DPPH solution at 517 nm; A_2_ represents the absorbance of the fermentation broth and ethanol at 517 nm; and A_0_ represents the absorbance of the ethanol and DPPH solution.

The ferric ion reducing antioxidant power (FRAP) was measured in accordance with the protocols provided in the total antioxidant capacity assay kit (Nanjing Jiancheng Bioengineering Institute, Nanjing, China).

### 2.6. Analysis of Microbial Community Structure

Samples after in vitro fermentation were centrifuged and DNA extracted to perform 16S rDNA sequencing. V3-V4 regions were amplified by PCR using 338F (5′-ACTCCTACGGGAGGCAGCA-3′) and 806R (5′-GGACTACHVGGGTWTCTAAT-3′). The amplification products were mixed in a certain proportion to form sub-libraries, which were sequenced by Illumina NovaSeq PE250 strategy. After quality control of raw sequencing data, DADA2 (Divisive Amplitude Denoising Algorithm 2, version 1.26.0) denoising method was used to construct OTU tables, followed by subsequent analysis of diversity, differences, species classification annotations, and others.

### 2.7. SCFAs Contents Detection During In Vitro Fermentation

The concentrations of short-chain fatty acids (SCFAs) in the fermentation broth were determined at 0, 6, 12, 24, and 48 h using a Shimadzu GC-2010 Plus gas chromatograph equipped with an SH-PolarD capillary column (0.25 mm × 0.25 μm × 30 m, Shimadzu, Kyoto, Japan). The brief procedure was as follows: The fermentation broth was centrifuged at 10,000 r/min for 10 min, and the supernatant was mixed with 45% phosphoric acid solution at a volume ratio of 15:1. After thorough mixing and a second centrifugation, the resulting supernatant was filtered through a 0.45 μm microporous membrane prior to gas chromatographic analysis. The chromatographic conditions were programmed with a maximum temperature below 450 °C.

### 2.8. Statistical Analysis

All experiments were independently performed in triplicate. Data were expressed as mean ± SEM. For comparisons among different groups at each time point, one-way ANOVA followed by Tukey’s post hoc test was used. For temporal changes within each group, two-way repeated-measures ANOVA was performed with treatment group and fermentation time as factors, followed by Tukey’s multiple comparisons test to identify significant differences between time points within each group. A significance threshold of *p* < 0.05 was applied. Graphs were generated using GraphPad Prism 10.1.2 and Origin 2024. Correlation analysis was conducted using MATLAB R2024b (The MathWorks, Inc., Natick, MA, USA).

## 3. Results and Discussion

### 3.1. Analysis of the Main Composition of PCPs from Different Growth Years

To evaluate the proportion of the polysaccharides in PCPs extracted from *P. cyrtonema* Hua, we detected the contents of total sugar, uronic acid and protein in PCPs. As shown in Table 1, total sugar made up more than 79% in PCPs, which is consistent with the findings of Zhao et al., who reported a total sugar content of 90.87 ± 1.77% in *P. cyrtonema* polysaccharides extracted without steam treatment [37], indicating the purity of PCPs was relatively high. The uronic acid content of FPP (40.60%) was significantly higher than that of TPP and EPP (*p* < 0.05). Given that uronic acid provides negatively charged carboxyl groups, its higher content may influence the conformational and colloidal behavior of polysaccharides, as demonstrated by Zhang et al., who found that uronic acid-rich green tea polysaccharides form gels via electrostatic interactions involving carboxyl groups [38]. In addition, TPP had the highest protein content (4.01%), which was significantly higher than that of FPP and EPP (*p* < 0.05). The presence of a small amount of protein in crude plant polysaccharides is common. Consistently, Tang et al. reported that physicochemical properties of pectic polysaccharides from bast fibers of ramie (RP) possessed the highest protein content (5.93%), and suggested that the presence of protein may affect the functional and bioactive potential of polysaccharides [39].

### 3.2. Changes in Basic Physicochemical Indicators During In Vitro Fermentation of PCPs from Different Growth Years

**Figure 1:**
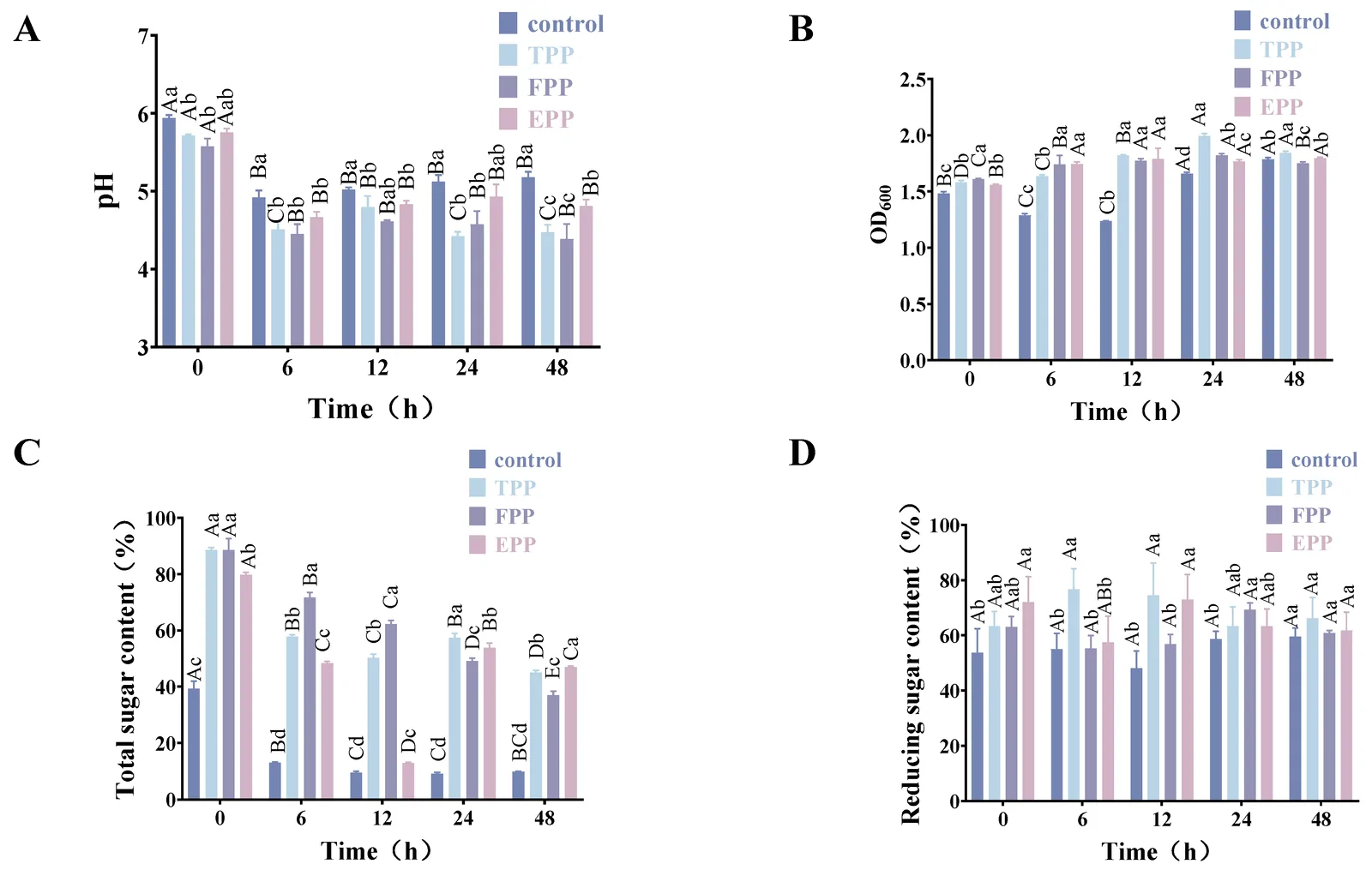
Changes in physicochemical indicators during the fermentation process of PCPs of different ages; (**A**) pH; (**B**) OD600; (**C**) total sugar content; (**D**) reducing sugar content. Different uppercase letters indicate significant differences in the same sample at different fermentation times; different lowercase letters indicate significant differences among different samples at the same fermentation time (*p* < 0.05). *P. cyrtonema* Hua polysaccharide from three-year-old plants (TPP); *P. cyrtonema* Hua polysaccharide from five-year-old plants (FPP); *P. cyrtonema* Hua polysaccharide from eight-year-old plants (EPP).

To study the in vitro fermentation properties of PCPs from different growth years, we analyzed changes in pH value, OD_600_, total sugar content, and reducing sugar content. The focus was to compare how gut microbiota utilize PCPs from different growth years. Gut microbiota could produce metabolites, including lactic acid and various SCFAs after fermentation to make the intestinal environment slightly acidic [40]. Dynamic changes in pH reflected the metabolic activity of gut microbiota and how they used the substrates. The results (Figure 1A) showed that the pH values in all three PCP groups decreased significantly during the first 6 h of fermentation (*p* < 0.05). The early decrease in pH might be due to acid production from probiotic metabolism [41], while the later slight increase might have resulted from further transformation or use of acidic substances in the system [42]. After 6 h, no significant changes in pH were observed within any group over the remaining fermentation period (*p* > 0.05). After 24 h and 48 h fermentation, the pH values of TPP and FPP were lower than that of EPP (*p* < 0.05).

The OD_600_ value reflects the growth status of the microbial community during fermentation. From Figure 1B, the OD_600_ values in all three PCP groups increased significantly from 0 h to 48 h (*p* < 0.05), and TPP showed the highest value at the endpoint (*p* < 0.05), suggesting TPP could promote more microorganisms to grow.

The change in total sugar content reflected how much the polysaccharides have been degraded. As shown in Figure 1C, the total sugar content in the control group was significantly lower than that in the sample group at each time point (*p* < 0.05), indicating PCPs could be the carbon source for gut microbiota. TPP decreased significantly within the first 12 h (a drop of 43.60%, *p* < 0.05), and the degradation of TPP matched its largest pH drop and highest initial OD value at the end of fermentation. In the FPP group, a significant decrease was observed throughout the fermentation (from 0 h to 48 h, *p* < 0.05), with a total reduction of 51.5%. In the EPP group, total sugar decreased significantly from 0 h to 6 h (*p* < 0.05) and then showed no significant changes thereafter (*p* > 0.05). Changes in reducing sugar content could further assess the degree of polysaccharide degradation. The reducing sugar content in the control groups was lower than that in the sample groups during fermentation (Figure 1D). This indicates that PCPs could be degraded and used by microorganisms. No significant difference in reducing sugar content was found among the three PCP groups at 0, 24, and 48 h (*p* > 0.05), while a significant difference appeared during the middle stage of fermentation (*p* < 0.05). Both at 6 and 12 h, the reducing sugar content of TPP was significantly higher than the other two groups (*p* < 0.05). All three groups showed a pattern of first increasing and then decreasing in reducing sugar content (*p* > 0.05), with different peak times, which was related to the release of reducing sugars from glycosidic bonds in the early fermentation stage, followed by their absorption and use by microorganisms. This finding is consistent with the conclusion of Zhu’s study on bitter melon polysaccharides [43]. In summary, all three PCPs were utilized by the gut microbiota under in vitro conditions, with TPP showing the most pronounced utilization among the three groups, as characterized by the lowest pH, the highest OD_600_, and the highest reducing sugar content at the end of fermentation.

### 3.3. Changes in the Structural Characteristics of PCPs During In Vitro Fermentation Across Different Growth Years

#### 3.3.1. Molecular Weight Changes

**Figure 2:**
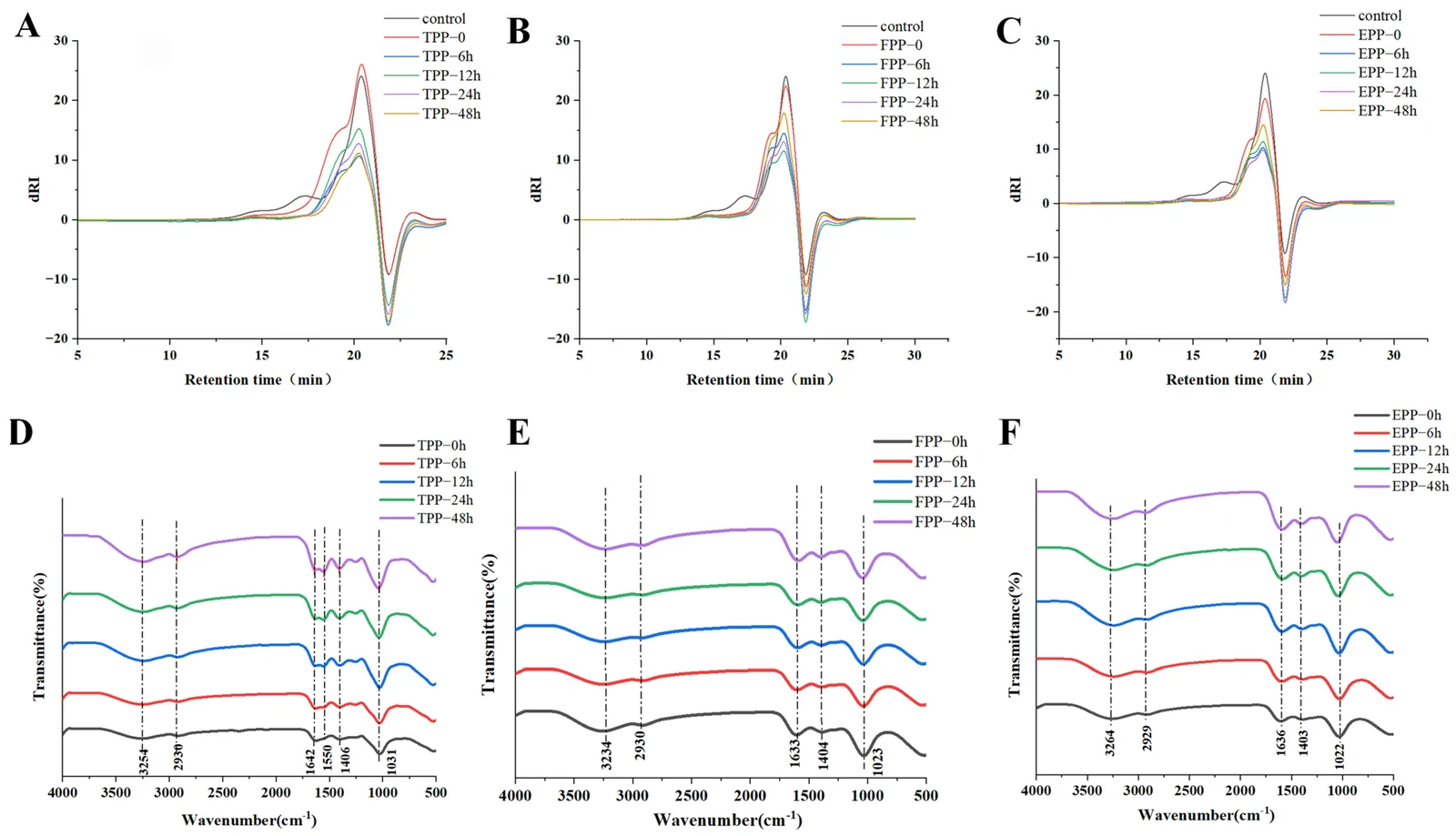
Changes in molecular weight and Fourier transform infrared (FT-IR) spectra of PCPs from different growth years during in vitro simulated fermentation. (**A**) TPP, (**B**) FPP, and (**C**) EPP: molecular weight distribution curves at different time points during fermentation; (**D**) TPP, (**E**) FPP, and (**F**) EPP: FT-IR spectra. Different letters indicate significant differences between groups (*p* < 0.05). *P. cyrtonema* Hua polysaccharide from three-year-old plants (TPP); *P. cyrtonema* Hua polysaccharide from five-year-old plants (FPP); *P. cyrtonema* Hua polysaccharide from eight-year-old plants (EPP).

To measure the molecular weight alterations of PCPs at different fermentation time points, we applied SEC-MALLS to detect the Mw and peak area. From Figure 2A–C and Table 2, we found the SEC peaks of PCPs kept relatively stable with rightward shifts in Mw profiles during the fermentation process; however, the Mw of PCPs showed fluctuations during fermentation, with a net decrease from 0 h to 48 h. In detail, after 48 h of fermentation, the Mw of TPP, FPP, and EPP decreased from 4.293 × 10^5^ kDa, 6.425 × 10^5^ kDa and 3.903 × 10^5^ kDa to 3.221 × 10^5^ kDa, 3.994 × 10^5^ kDa and 2.906 × 10^5^ kDa, respectively. Additionally, the dispersibility coefficients of PCPs were all greater than 1, suggesting PCPs may be degraded by gut microbiota into lower molecular weight fragments. Similar studies observed that the molecular weight of *Pyracantha fortuneana* polysaccharide and longan polysaccharide also decreased to generate smaller ones during fermentation [44,45]. The degradation of polysaccharides during fermentation was largely ascribed to the enzyme hydrolysis, such as various glycosidases belonging to the Glycoside Hydrolases (GH) family in the microorganisms in the large intestine [46,47]. The increase in Mw observed at 6–12 h may be attributable to potential overlap between polysaccharide peaks and components derived from the culture medium [48]; therefore, these intermediate values should be interpreted with caution and require further verification.

#### 3.3.2. FT-IR Spectral Analysis

Fourier transform infrared spectroscopy showed (Figure 2D–F) that after fermentation, all three PCPs from different growth years still had typical polysaccharide absorption peaks and the main chain structures did not change significantly. However, the intensities of the absorption peaks at 1642 cm^−1^ and 1406 cm^−1^ appeared to increase after fermentation, suggesting a relative rise in acidic group content. However, as FT-IR analysis is semi-quantitative, further quantitative analysis is needed to confirm this observation. Take TPP as an example; its main absorption peaks were as follows: 3254 cm^−1^ (O-H stretching vibration), 2930 cm^−1^ (C-H symmetric stretching vibration of -CH_2_), 1642 cm^−1^ and 1406 cm^−1^ (characteristic vibration of glycolic acid C=O), and 1031 cm^−1^ (asymmetric stretching vibration of C-O-C in the pyranose ring). The spectra at all time points were generally similar. This confirmed that the fermentation process did not damage the main chain structure of the polysaccharide.

#### 3.3.3. Monosaccharide Composition Changes

This study measured changes in monosaccharide content in the culture medium during fermentation. The results showed that the types of monosaccharides in the three PCPs were similar before and after fermentation (Table 3). These included arabinose, glucose, rhamnose, galactose, xylose, glucuronic acid, galacturonic acid, and fucose. After fermentation, glucose contents decreased the most in all three groups (by 25.88%, 24.06%, and 27.10%, respectively), indicating that glucose was the preferred carbon source for microorganisms. Xylose content increased the most (by 15.85%, 12.00%, and 12.05% for TPP, FPP and EPP, respectively). In addition, the contents of arabinose, rhamnose, and galactose all increased, while fucose content decreased. This suggests that the intestinal microbiota not only have polysaccharide hydrolases but may also contain various isomerases that participate in the conversion and use of monosaccharides. After fermentation, FPP showed a slight increase in galacturonic acid content, consistent with the contents of uronic acid in FPP. TPP and EPP exhibited increased glucuronic acid contents, while FPP showed a slight decrease. The variations in monosaccharide release patterns among different polysaccharides depended on their structural configurations and fermentation conditions. For instance, Xu et al. observed decreased galactose and galacturonic acid in fermented *Astragalus* polysaccharides, suggesting substrate-specific microbial metabolism [49].

### 3.4. Evaluation of the Antioxidant Activity of PCPs from Different Growth Years In Vitro

To evaluate the dynamic changes in antioxidant activity of PCPs from different growth years during in vitro fermentation, this study measured the ABTS^+^ and DPPH scavenging abilities, and FRAP reducing abilities at different time points.

**Figure 3:**
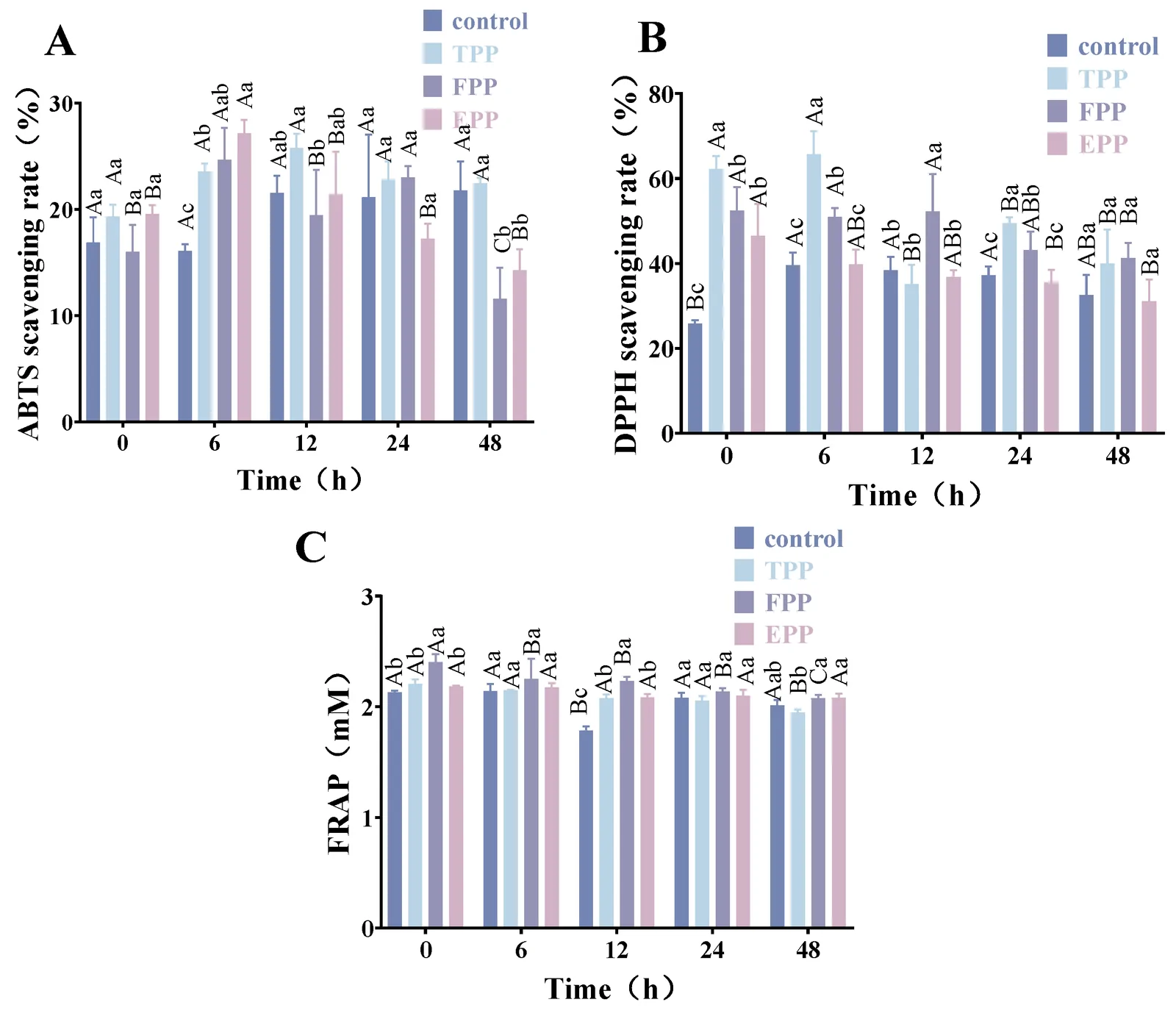
Antioxidant activities at different time points during the glycolysis of PCPs of different ages; (**A**) ABTS scavenging rate; (**B**) DPPH scavenging rate; (**C**) ferric ion radical scavenging rate. Different uppercase letters indicate significant differences in the same sample at different fermentation times; different lowercase letters indicate significant differences among different samples at the same fermentation time (*p* < 0.05). *P. cyrtonema* Hua polysaccharide from three-year-old plants (TPP); *P. cyrtonema* Hua polysaccharide from five-year-old plants (FPP); *P. cyrtonema* Hua polysaccharide from eight-year-old plants (EPP).

ABTS radical scavenging assay is one of the most widely employed methods for evaluating the in vitro antioxidant capacity of polysaccharides. As shown in Figure 3A, in the FPP and EPP groups, the ABTS^+^ scavenging activity increased significantly from 0 h to 6 h (*p* < 0.05), followed by a plateau phase with no significant changes through 24 h, and then decreased significantly from 24 h to 48 h (*p* < 0.05). In contrast, no significant changes were observed in the TPP group throughout the fermentation period (*p* > 0.05). At 6 h of fermentation, the ABTS^+^ scavenging ability of TPP was significantly lower than that of the EPP group (*p* < 0.05), while at 48 h, it was obviously higher than that of the FPP and EPP groups (*p* < 0.05). It demonstrated that the antioxidant activity of EPP was quickly presented in the early stage of fermentation; in contrast, TPP responded later, probably attributing to TPP being exposed to more hydroxyl groups and porous configurations to enhance radical neutralization at a later stage [50].

DPPH radical scavenging assay is a widely used method for evaluating the hydrogen-donating capacity of natural antioxidants. The DPPH assay results (Figure 3B) showed that at 0, 6, and 24 h of fermentation, the TPP clearance rate was significantly higher than that of FPP and EPP, while FPP exhibited the highest clearance rate at 12 h (*p* < 0.05). As a result, after 12 h, FPP was significantly higher than TPP (*p* < 0.05). After fermentation ended, the clearance rates of TPP, FPP, and EPP were all decreased (*p* < 0.05), suggesting that the overall fermentation process reduced DPPH scavenging ability, which is in agreement with previous studies on *Sargassum pallidum* polysaccharide, possibly due to conformational alterations [51]. Among the three, TPP showed relatively superior performance over the others.

The total reducing power of different PCPs before and after fermentation was also measured by the FRAP assay. The FRAP results showed that the iron reduction capacity of PCPs slightly decreased the fermentation overall (Figure 3C), and FPP performed better than the other two at 0 and 12 h (*p* < 0.05).

The structural differences among PCPs derived from different growth years were the primary determinants of their distinct antioxidant activities. During in vitro simulated fermentation, FPP exhibited a higher molecular weight and greater uronic acid content, which contributed to its superior ferric reducing power compared to TPP and EPP. Additionally, TPP possessed slightly higher levels of total sugar and reducing sugar than FPP and EPP, thereby conferring better overall antioxidant performance.

### 3.5. Effects of In Vitro Fermentation of PCPs from Different Growth Years on the Diversity and Community Structure of the Gut Microbiota

**Figure 4:**
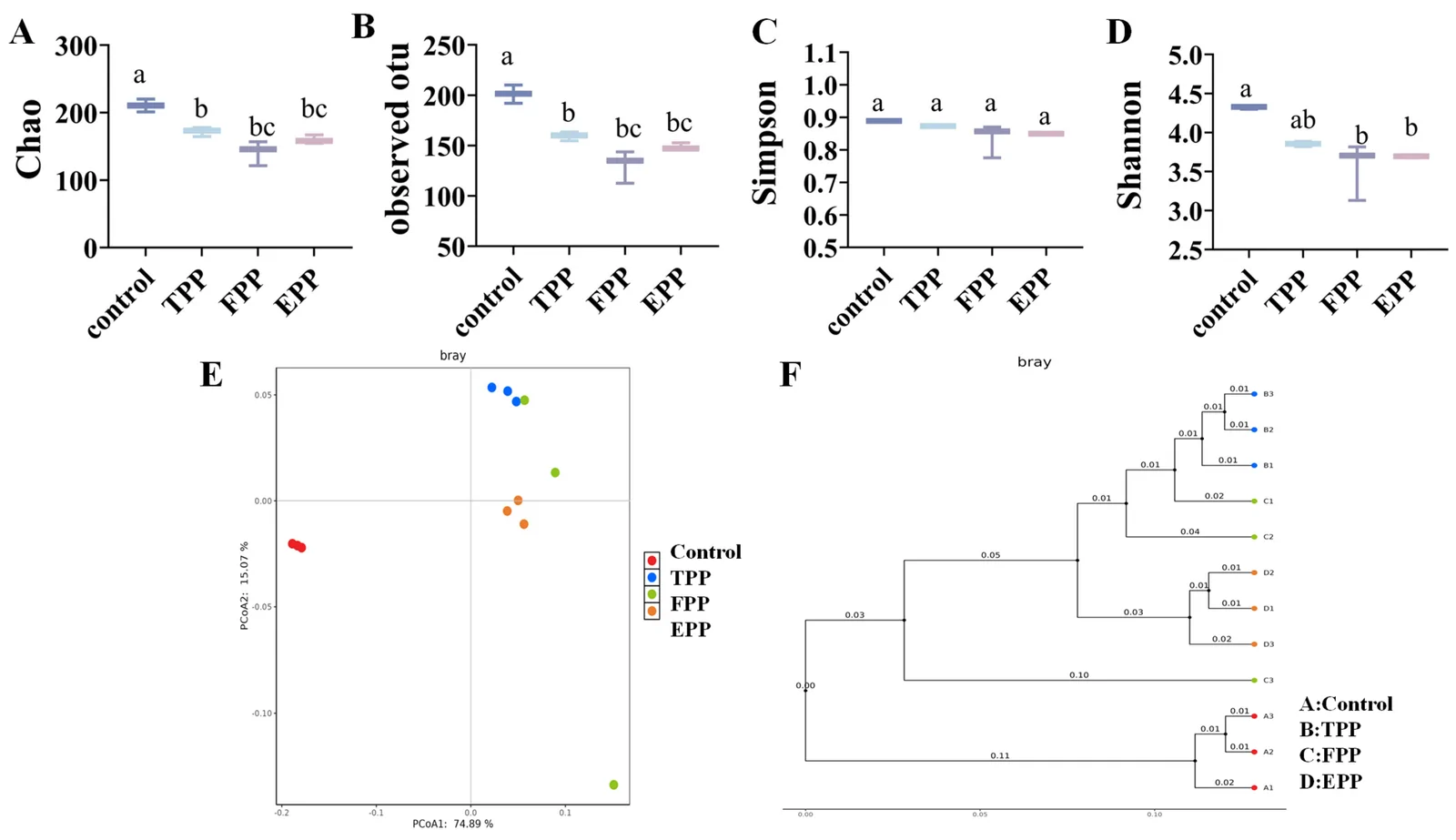
Gut microbial diversity; (**A**) Chao1 index; (**B**) operational taxonomic unit index analysis; (**C**) Simpson index; (**D**) Shannon index α diversity analysis; (**E**,**F**) Beta diversity. Different letters indicate significant differences between groups (*p* < 0.05). *P. cyrtonema* Hua polysaccharide from three-year-old plants (TPP); *P. cyrtonema* Hua polysaccharide from five-year-old plants (FPP); *P. cyrtonema* Hua polysaccharide from eight-year-old plants (EPP).

This study performed α-diversity analysis to assess the richness and diversity of the gut microbiota. The Chao1 and Observed species indices were used to represent microbial richness. The Simpson and Shannon indices were used to reflect diversity. The results showed that compared with the control group, microbial richness in all three PCP groups markedly decreased (*p* < 0.05), and microbial diversity was lower (Figure 4A–C). This observation suggested that PCPs might reduce overall diversity and richness by promoting the growth of specific bacteria. Previous in vitro studies have consistently demonstrated that intervention with various polysaccharides, including almond polysaccharide AP-1 [52], *Polygonatum cyrtonema* polysaccharide [53], and four varieties of Tibetan tea polysaccharides [54], reduced gut microbial richness and diversity during in vitro fermentation by enriching beneficial bacteria such as *Megamonas* and *Bifidobacterium*. Beta diversity reflects the uniqueness of species within a community. Principal coordinate analysis (PCoA) showed that PCoA1 (74.89%) and PCoA2 (15.07%) together explained 89.96% of the variation. Significant differences were found between the control group and PCPs from different growth years (Figure 4E,F), further demonstrating that PCPs changed the composition of the gut microbiota.

**Figure 5:**
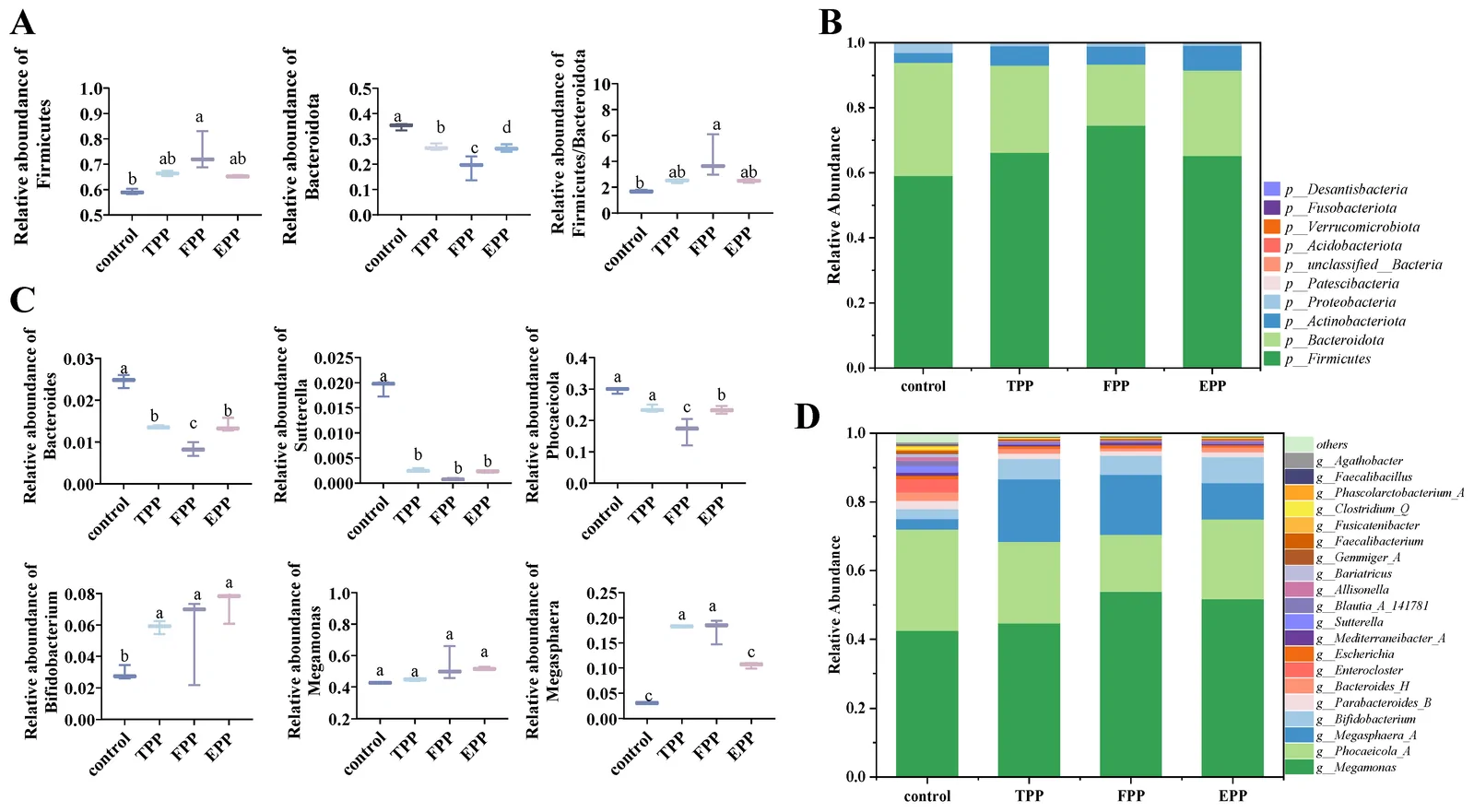
Composition of the gut microbiota. (**A**) Relative abundance of samples at the phylum level; (**B**) heatmap of species distribution at the phylum level; (**C**) relative abundance of samples at the genus level; (**D**) heatmap of species distribution at the genus level. Different letters indicate significant differences between groups (*p* < 0.05). *P. cyrtonema* Hua polysaccharide from three-year-old plants (TPP); *P. cyrtonema* Hua polysaccharide from five-year-old plants (FPP); *P. cyrtonema* Hua polysaccharide from eight-year-old plants (EPP).

The composition of gut microbial communities was as shown in Figure 5A,B. At the phylum level, Firmicutes, Bacteroidota, Actinobacteriota, and Proteobacteria were the four dominant phyla of the human gut microbiota. PCPs could enrich Firmicutes and downregulate Bacteroidota level, especially for FPP. This is consistent with a previous in vitro fermentation study on the polysaccharide of *Ziziphus jujuba* cv. *Pozao*, which promoted the growth of Firmicutes while reducing the abundance of Bacteroidetes and Proteobacteria [55]. Further analysis showed that the Firmicutes/Bacteroidetes ratio (F/B ratio) in all three PCP groups was higher than that in the control group, suggesting that PCPs intervention could increase the F/B ratio, which could in turn promote the absorption of food energy by the gut microbiota [56]. In summary, PCPs modulated the composition of intestinal microorganisms at the phylum level with an increasing F/B ratio.

Figure 5C,D illustrate changes in the relative abundance of the top 20 genera among different groups. Compared with the blank group, the three PCP groups exhibited a trend toward enriching beneficial genera including *Megasphaera* and *Bifidobacterium*, while inhibiting potentially pathogenic genera including *Sutterella*, *Escherichia* and *Phocaeicola*. *Megasphaera* can use lactic acid in the human intestine to produce valeric acid [57] and promote the production of SCFAs such as butyric acid, thereby improving the intestinal environment. The enrichment of *Megasphaera* was most significant in TPP and FPP, with relative abundances rising to 18.29% and 17.53%, respectively. *Bifidobacterium* can break down complex carbohydrates, promote energy metabolism, and produce beneficial metabolites for the host, such as SCFAs [58]. For *Bifidobacterium*, the promoting effect of EPP was the strongest (7.48%), higher than that of FPP (5.88%) and TPP (5.50%), but without significant differences. Among the three groups, FPP had the highest relative abundance of *Megamonas* (53.93%), compared to TPP (44.76%) and EPP (51.79%). *Megamonas* is known for rapid proliferation, which can ferment carbohydrates to produce acetic acid, propionic acid, lactic acid, and other metabolites. The trend for *Bacteroides* was consistent with the results observed at the phylum level for Bacteroidetes. As shown in Figure 5C, the relative abundances of *Phocaeicola* in the TPP, FPP, and EPP groups were significantly lower than that in the control group (29.55%), in which FPP showed the strongest inhibitory effect on *Phocaeicola* (*p* < 0.05). Compared with the blank group (1.90%), the abundance of *Sutterella* was significantly reduced in all three treatment groups. Among them, FPP showed the strongest inhibitory effect, with abundance dropping to 0.08%. *Sutterella* has been identified as a pathogenic gut bacterium associated with ulcerative colitis and childhood obesity [51]. Consistently, a study by Xue Han et al. reported that the polysaccharide from *Ziziphus Jujuba* cv. *Pozao* significantly suppressed the abundance of *Sutterella* following in vitro fermentation [55]. In summary, all three PCPs showed prebiotic potential via modulating gut microbiota under in vitro fermentation conditions, but each exhibited distinct advantages in modulating specific bacterial genera, with no consistent growth-year-dependent trend observed.

### 3.6. Effects of PCPs from Different Growth Years on Intestinal SCFAs During In Vitro Fermentation

**Figure 6:**
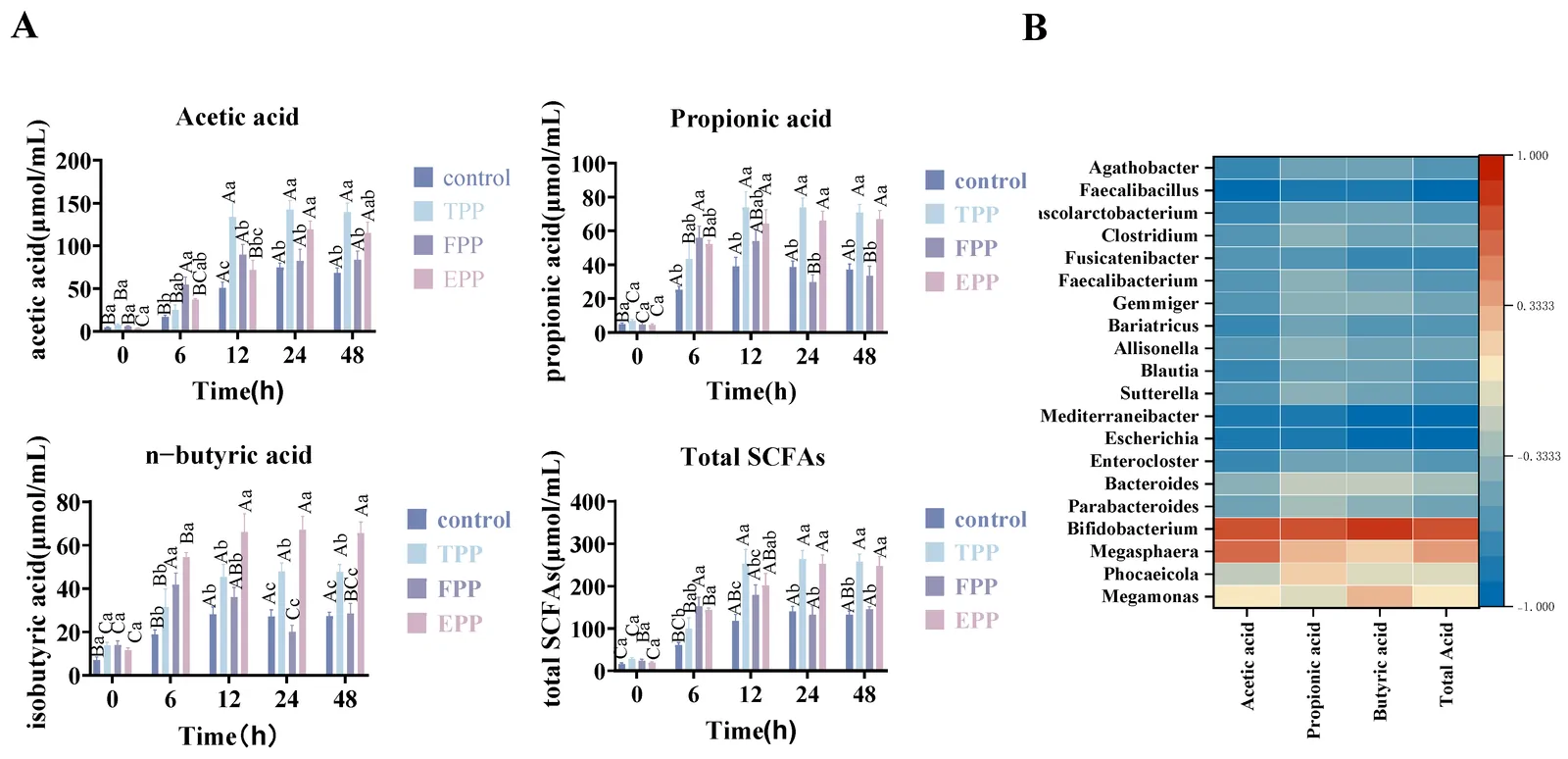
Short-chain fatty acids. (**A**) Contents of acetic acid, propionic acid, n-butyric acid, and total acids; (**B**) correlation heatmap between gut microbiota at the genus level and short-chain fatty acids. Different uppercase letters indicate significant differences in the same sample at different fermentation times; different lowercase letters indicate significant differences among different samples at the same fermentation time (*p* < 0.05). *P. cyrtonema* Hua polysaccharide from three-year-old plants (TPP); *P. cyrtonema* Hua polysaccharide from five-year-old plants (FPP); *P. cyrtonema* Hua polysaccharide from eight-year-old plants (EPP).

As the dominating metabolite of gut microbiota, SCFAs are the key indicator for evaluating the probiotic activity of polysaccharides after fermentation. SCFAs play an important role in maintaining intestinal homeostasis [59]. Acetic acid, propionic acid, and butyric acid were the main SCFAs produced in the TPP, FPP, EPP, and control groups. Acetate promotes anorexigenic gut hormone Peptide YY secretion by increasing intracellular cAMP levels in intestinal endocrine cell lines, which helps regulate appetite and maintain energy balance [60]. Propionic acid can lower serum cholesterol, prevent diet-induced obesity, and improve tissue insulin sensitivity [61]. Butyric acid serves as an energy source for colonic epithelial cells and also can reduce inflammatory responses and help improve metabolic syndrome [62]. As shown in Figure 6A, the total SCFA concentrations in all three PCP groups increased significantly during the initial fermentation stage. Specifically, TPP and EPP exhibited continuous significant increases from 0 h to 12 h (*p* < 0.05), followed by stabilization with no significant changes thereafter (*p* > 0.05). In contrast, FPP showed a significant increase only from 0 h to 6 h (*p* < 0.05), with no significant changes observed after 6 h (*p* > 0.05). At the endpoint of fermentation, total SCFA levels in TPP and EPP were significantly higher than those in the control and FPP groups, whereas FPP exhibited a rapid increase in total SCFAs at 6 h. Similar patterns were observed for acetic acid, propionic acid, and n-butyric acid. Collectively, TPP and EPP showed greater SCFA production than FPP at the endpoint of fermentation, with TPP favoring acetic acid and propionic acid generation (propionic acid peaked at 73.89 μmol/mL, 12 h) and EPP favoring n-butyric acid production (67.08 μmol/mL, 24 h). FPP showed the lowest acetic and butyric acid levels but an early propionic acid peak (55.81 μmol/mL, 6 h), indicating transient metabolic regulation, which may be related to its higher glucuronic acid and galacturonic acid contents [63]. In contrast, TPP with a comparatively lower initial molecular weight was more favorable for SCFA production [59]. Collectively, both TPP and EPP demonstrated potent SCFA-producing potential, yet the levels of individual SCFAs varied, which could be attributed to the differences in their monosaccharide composition ratios.

We further analyzed the correlation between the relative abundance of gut microbiota at the genus level and SCFA levels to identify potential microbiota involved in SCFA production. As shown in Figure 6B, *Megasphaera* and *Bifidobacterium* were positively correlated with acetic acid, propionic acid, and n-butyric acid. In addition, *Megamonas* was positively correlated with acetic acid and n-butyric acid. *Bifidobacterium* ferments carbohydrates to produce acetic acid and lactic acid as main end products [58], while *Megasphaera* is known for butyric acid production, and *Megamonas* generates acetic acid and propionic acid through fermentation [64]. These results suggested that PCP may promote SCFAs production by enriching bacterial communities such as *Megamonas*, *Megasphaera*, and *Bifidobacterium* under in vitro conditions. From Figure 5C, although FPP showed better performance to enrich *Megamonas*, *Megasphaera*, and *Bifidobacterium* than TPP, the much lower levels of SCFAs observed suggest that the functional capacity of the microbial community is not only determined by abundance but also influenced by the microenvironment. The lower pH in the environment of the FPP group probably could inhibit the activity of enzymes in *Megasphaera* that switch the pathway of SCFAs production to other metabolic pathways, such as the lactic acid production [65,66]. Furthermore, *Phocaeicola* was negatively correlated with acetic acid and n-butyric acid. Sixteen other bacteria, including *Parabacteroides*, *Bacteroides*, *Enterocloster*, and *Sutterella*, also show negative correlations with SCFAs. This demonstrated that PCP might help optimize intestinal SCFA levels by inhibiting these bacteria.

## 4. Conclusions

This study systematically characterized the in vitro fermentation characteristics, gut microbiota-modulating effects, and SCFA production profiles of PCPs derived from three different growth years. The three PCPs exhibited notable differences in uronic acid content, molecular weight distribution, and in vitro antioxidant capacity. All three PCPs enhanced SCFAs production through differential modulation of gut microbiota composition. Specifically, TPP demonstrated the strongest enrichment of *Megasphaera* and sustained propionic acid production, reflecting a balanced gut regulatory capacity. FPP exhibited the most complete degradation and the earliest propionic acid peak, but produced the lowest levels of acetic acid and butyric acid, indicating only short-term metabolic regulation. In contrast, EPP achieved the most balanced SCFA profile through synergistic enrichment of multiple bacterial genera, making it suitable for long-term maintenance of intestinal health. In summary, structural differences arising from growth years led to distinct fermentation characteristics and prebiotic efficacies among the three PCPs, with no consistent enhancement in probiotic effects observed as growth years increased. From a cost-effectiveness perspective, TPP represents a promising and economical candidate for the development of functional foods, warranting further in vivo investigation.

## Figures and Tables

**Table 1:** Contents of total sugars, uronic acids, and proteins in PCPs from different years.

	Total Sugar Content (%)	Uronic Acid Content (%)	Protein Content (%)
TPP	88.57 ± 0.85 ^a^	22.97 ± 0.18 ^c^	4.01 ± 1.48 ^a^
FPP	88.54 ± 4.06 ^a^	40.60 ± 0.37 ^a^	0.62 ± 0.52 ^b^
EPP	79.75 ± 0.92 ^b^	26.28 ± 0.17 ^b^	1.67 ± 0.42 ^b^

*P. cyrtonema* Hua polysaccharide from three-year-old plants (TPP); *P. cyrtonema* Hua polysaccharide from five-year-old plants (FPP); *P. cyrtonema* Hua polysaccharide from eight-year-old plants (EPP). Different letters indicate significant differences between groups (*p* < 0.05).

**Table 2:** Changes in molecular weight during fermentation of PCPs of different ages.

Time (h)	Mw (kDa)		
	TPP	FPP	EPP
0	4.293 × 10^5 ABab^	6.425 × 10^5 Ba^	3.903 × 10^5 Bb^
6	4.012 × 10^5 ABb^	4.242 × 10^5 Cb^	7.701 × 10^5 Aa^
12	4.886 × 10^5 Ab^	9.864 × 10^5 Aa^	9.809 × 10^5 Aa^
24	4.432 × 10^5 ABb^	8.427 × 10^5 Aa^	2.184 × 10^5 Bb^
48	3.221 × 10^5 Bab^	3.994 × 10^5 Ca^	2.906 × 10^5 Bb^

*P. cyrtonema* Hua polysaccharide from three-year-old plants (TPP); *P. cyrtonema* Hua polysaccharide from five-year-old plants (FPP); *P. cyrtonema* Hua polysaccharide from eight-year-old plants (EPP). Different uppercase letters indicate significant differences in the same sample at different fermentation times; different lowercase letters indicate significant differences among different samples at the same fermentation time (*p* < 0.05).

**Table 3:** Monosaccharide composition of three PCPs before and after fermentation.

Monosaccharides (%)	TPP-0 h	TPP-48 h	FPP-0	FPP-48 h	EPP-0	EPP-48 h
Ara	11.85 ^b^	15.45 ^a^	9.9 ^b^	12.64 ^a^	9.66 ^b^	18.45 ^a^
Glu	35.62 ^a^	9.74 ^b^	33.29 ^a^	9.23 ^b^	38.08 ^a^	10.98 ^b^
Rha	3.89 ^b^	7.45 ^a^	4.5 ^b^	7.48 ^a^	2.31 ^b^	7.97 ^a^
Gal	17.24 ^b^	22.23 ^a^	15.71 ^b^	21.4 ^a^	17.29 ^b^	25.65 ^a^
Xyl	21.89 ^b^	37.74 ^a^	18.52 ^b^	30.52 ^a^	17.48 ^b^	29.53 ^a^
GalA	2.11 ^a^	1.3 ^b^	9.11 ^a^	9.94 ^a^	2.65 ^a^	1.73 ^b^
GlcA	0.63 ^b^	1.13 ^a^	0.96 ^a^	0.7 ^b^	0.76 ^a^	0.9 ^a^
L-Fuc	6.77 ^a^	4.95 ^b^	9.01 ^a^	8.08 ^a^	11.77 ^a^	4.79 ^b^

*P. cyrtonema* Hua polysaccharide from three-year-old plants (TPP); *P. cyrtonema* Hua polysaccharide from five-year-old plants (FPP); *P. cyrtonema* Hua polysaccharide from eight-year-old plants (EPP). Different letters denote significant differences between fermentation time points (*p* < 0.05).

## Data Availability

The original contributions presented in this study are included in the article. Further inquiries can be directed to the corresponding authors.
